# Association Between Nonmotor Symptoms and Nonadherence to Medication in Parkinson's Disease

**DOI:** 10.3389/fneur.2020.551696

**Published:** 2020-10-19

**Authors:** Sarah Mendorf, Otto W. Witte, Hannah Zipprich, Tino Prell

**Affiliations:** ^1^Department of Neurology, Jena University Hospital, Jena, Germany; ^2^Centre for Healthy Ageing, Jena University Hospital, Jena, Germany

**Keywords:** adherence, antiparkinson drugs, non-motor symptom, anxiety, depression

## Abstract

**Background:** Nonadherence to medication is a common and serious issue in the treatment of patients with Parkinson's disease (PD). Among others, distinct nonmotor symptoms (NMS) were found to be associated with nonadherence in PD. Here, we aimed to confirm the association between NMS and adherence.

**Methods:** In this observational study, the following data were collected: sociodemographic data, the German versions of the Movement Disorder Society-sponsored revision of the unified Parkinson's disease rating scale for motor function (MDS-UPDRS III), Hoehn and Yahr (H&Y) stage, levodopa equivalent daily dose (LEDD), Becks depression inventory II (BDI-II), nonmotor symptoms questionnaire (NMSQ), and the Stendal adherence to medication score (SAMS).

**Results:** The final sample included 137 people with PD [54 (39.4%) females] with a mean age of 71.3 ± 8.2 years. According to SAMS, 10.9% of the patients were fully adherent, 73% were moderately nonadherent, and 16.1% showed clinically significant nonadherence. Nonadherence was associated with LEDD, BDI-II, education level, MDS-UPDRS III, and the NMSQ. The number of NMS was higher in nonadherent patients than in adherent patients. In the multiple stepwise regression analysis, the items 5 (constipation), 17 (anxiety), and 21 (falls) predicted nonadherence to medication. These NMSQ items also remained significant predictors for SAMS after correction for LEDD, MDS-UPDRS III, BDI-II, age, education level, gender, and disease duration.

**Conclusion:** Our study, in principle, confirms the association between NMS burden and nonadherence in PD. However, in contrast to other clinical factors, the relevance of NMSQ in terms of nonadherence is low. More studies with larger sample sizes are necessary to explore the impact of distinct NMS on adherence.

## Introduction

Parkinson's disease (PD) is a common neurodegenerative disorder predicted to increase in prevalence as the population ages (1). It is characterized by motor symptoms that include tremor, rigidity, and bradykinesia. However, a plethora of nonmotor symptoms (NMS) can occur and contribute to clinical heterogeneity. These NMS, such as neurobehavioral disorders, cognitive impairment, gastrointestinal dysfunction, bladder dysfunction, or sexual dysfunction, are, by all accounts, very common in individuals with PD and contribute to poor quality of life (QoL) (2).

Nonadherence to medication is a major issue in this chronic disorder that results in reduced QoL and higher readmission rates (3, 4). It represents a financial burden to the health system and reduces the benefit from pharmacotherapy (5–9). Many reasons exist why people with PD do not or cannot follow instructions given for prescribed medical treatments. Several epidemiological and clinical factors were found to be associated with nonadherence to medication in PD, such as younger age, education level, marital status, depression and anxiety, poor cognition, longer disease duration, and regimen complexity (3, 4, 6, 10–17). Recently, Straka et al. studied the relationship between NMS and adherence in 124 subjects with PD recruited from movement disorder outpatient departments of university hospitals in Slovakia (18). Here, adherence scores [eight-item Morisky medication adherence scale (MMAS-8)] positively correlated with disease duration, health-related QoL, depression, frequency, and severity of NMS [NMS scale (NMSS)], and motor and nonmotor complications. However, in their linear regression, only NMSS scale predicted MMAS-8. In particular, excessive daytime sleepiness, anhedonia, and forgetfulness (items 3, 12, and 18 of NMSS) predicted worse adherence to PD medication. This study was limited by the selection of patients who take a minimum of three daily doses of PD medication. It was also surprising that depression was not an independent predictor of adherence beside the NMSS. Therefore, we aimed to replicate the association between NMS and adherence by using two different questionnaires for NMS and adherence.

## Methods

### Participants and Assessments

The study was approved by the local ethics committee of the Jena University Hospital, and written informed consent was obtained from all patients. Data from patients with PD were consecutively collected from the outpatient clinic of the Department of Neurology at the Jena University Hospital. Inclusion criteria include PD diagnosis according to Movement Disorder Society (MDS) diagnosis criteria, which was made by a movement disorder specialist (TP), while exclusion criteria include cerebrovascular disorders, delirium, unable to complete a questionnaire, and PD dementia [Montreal cognitive assessment (MoCA) <21 points]. All tests and assessments were conducted during the medication ON phase. Demographic data include age, gender, marital status, employment status, and level of education (high, German Abitur or University; low, German Realschule or General Certificate of Secondary Education, German Hauptschule or no school). Several clinical variables were recorded using the validated German questionnaires and assessments: MDS-sponsored revision of the Unified Parkinson's disease rating scale for motor function (MDS-UPDRS III), revised nonmotor symptoms questionnaire (NMSQ), Hoehn and Yahr (H&Y) staging, and levodopa equivalent daily dose (LEDD). Nonmotor symptoms questionnaire is a comprehensive single-page, self-administered 30-item assessment for a diverse range of NMS in PD. It has good patient acceptability (19, 20), and in contrast to NMSS, it is not designed to assess the severity or frequency of NMS. Montreal cognitive assessment was used to assess cognition (21) and Becks depression inventory II (BDI-II) to quantify depressive mood. The German Stendal adherence to medication score (SAMS) was used to assess adherence. It includes 18 questions forming a cumulative scale (0–72), with 0 indicating complete adherence and 72 complete nonadherence (22). The sum of scores ranged from 0 (fully adherent) to 72 (fully nonadherent). Moreover, it allows the assessment of three common reasons for/clusters of nonadherence: modification of medications, forgetting to take the medications, and lack of knowledge about the medications (23). The whole copy of SAMS is available online (CC BY NC 3.0 license; https://data.mendeley.com/datasets/ny2krr3vgg/1) (24).

The total number of patients recruited for the study was 140; three cases had incomplete or missing data and thus were excluded.

### Statistical Analysis

Statistics were performed with the statistical software SPSS 25.0 (SPSS Inc., Chicago IL, USA). Data were first analyzed by means of descriptive statistics: means, standard deviations, median, interquartile range, frequencies, and percentages. Data were checked for normality using the Shapiro–Wilk test.

There is no established threshold to determine nonadherence. A cut-off point of 0.80 was found to be reasonable and valid in stratifying adherent and nonadherent patients based on predicting subsequent hospitalization across several highly prevalent chronic diseases. Therefore, it is generally considered that suboptimal adherence becomes clinically significant when <80% of prescribed medication is taken (14, 25). In our study, the highest 20% of all SAMS scores were categorized as nonadherent. This leads to a study- and sample-specific SAMS cutoff of 13 points for a clinically significant nonadherence. The patients were then categorized into (a) fully adherent (SAMS = 0), (b) moderately nonadherent (SAMS 1–12), and (c) nonadherent (SAMS ≥ 13). Spearman correlation was used to test the correlation between NMSQ and SAMS total score. Multiple forward stepwise linear regression analyses were subsequently performed to ascertain independent predictors of SAMS. The significance level for variables entering into the linear regression model was set at 0.2 and removing from the model at 0.4. The independent variables were derived from the literature and are presented in the corresponding tables (3, 4, 6, 10–16). The level of statistical significance was set at *P* < 0.05.

### Declaration of Sources of Funding

This work was supported by a BMBF (Bundesministerium für Bildung und Forschung) grant to Tino Prell (01GY1804).

## Results

The final sample included 137 people with PD (54 [39.4%] female) with a mean age of 71.3 ± 8.2 years. Majority of the patients were married and completed middle or high school education. Detailed clinical data are presented in Table 1. According to SAMS, 10.9% (*n* = 15) of the patients were fully adherent (SAMS = 0), 73% (*n* = 100) were moderately nonadherent (SAMS 1–12), and 16.1% (*n* = 22) showed clinically significant nonadherence (SAMS ≥ 12).

**Table 1:** Clinical and demographic characteristics.

		***n***	**%**
Sex	Female	54	39.4
	Male	83	60.6
Education	Low	73	56.6
	High	56	43.4
Marital status	Widowed, divorced, separated	28	21.7
	Married	96	74.4
	Single	5	3.9
		**Mean**	**SD**
Age (years)		71.3	8.2
Number of medications per day		7.7	5.0
Levodopa equivalent daily dose (LEDD)		651.1	433.2
Disease duration (years)		9.0	6.6
Hoehn and Yahr stage (median, IQR)		3.0	1.0
Movement Disorder Society-sponsored revision of the unified Parkinson's disease rating scale (MDS-UPDRS) III		27.1	14.9
Nonmotor symptoms questionnaire (NMS-Q)		10.0	4.9
Montreal cognitive assessment (MoCA)		24.7	2.6
Becks depression inventory (BDI)-II		11.1	8.1
Stendal adherence to medication score (SAMS)		7.2	6.8

**Figure 1:**
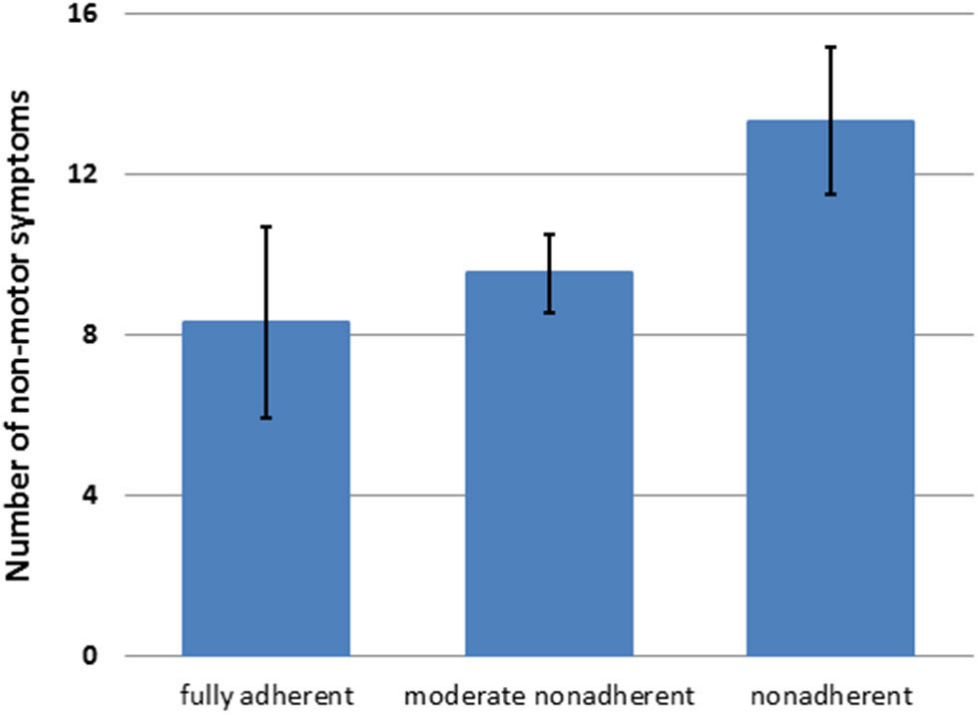
Number of nonmotor symptoms [nonmotor symptoms questionnaire (NMSQ) total score] in patients with different degrees of nonadherence (mean + 95%CI).

In the multiple linear regression analysis, SAMS was associated with LEDD, BDI-II, education level, MDS-UPDRS III, and NMSQ (corrected *R*^2^ = 0.23, *P* < 0.001, Table 2). Accordingly, the number of NMS increased with the degree of nonadherence (Figure 1).

**Table 2:** Multiple linear regression models for the prediction of Stendal adherence to medication score (SAMS).

Predictor^*^	**Unstandardized coefficients**		**Standardized coefficients**	***t***	***P*-value**
	***b***	**SE**	**β**		
**PREDICTION OF SAMS BY SEVERAL CLINICAL FACTORS**					
Constant	−0.697	1.699		−0.41	0.682
LEDD	0.005	0.001	0.43	3.64	<0.001
BDI-II	0.200	0.083	0.188	2.41	0.017
Education level (low)	−2.293	1.053	0.154	−2.28	0.031
MDS-UPDRS III	0.075	0.036	0.138	2.10	0.041
NMSQ	0.200	0.119	0.091	1.70	0.97
**Predictor**^**#**^	**Unstandardized coefficients**		**Standardized coefficients**	***t***	***p***
	***b***	**SE**	**β**		
**PREDICTION OF SAMS BY DISTINCT NMSQ ITEMS**					
Constant	13.36	1.40		9.54	<0.001
NMS item 5 = 0	−3.74	1.41	0.499	−3.28	0.001
NMS item 21 = 0	−2.64	1.09	0.268	−2.40	0.018
NMS item 17 = 0	−2.89	1.28	0.233	−2.24	0.027
The independent variables were NMSQ item 1 (hypersalivation), 5 (constipation), 13 (loss of interest), 15 (problems concentration), 17 (anxiety) and 21 (falls)					
**Predictor**^**§**^	**Unstandardized coefficients**		**Standardized coefficients**	***t***	***p***
	***b***	**SE**	**β**		
**PREDICTION OF SAMS BY CLINICAL FACTORS AND DISTINCT**					
**NMSQ ITEMS**					
Constant	8.05	2.21			<0.001
LEDD	0.005	0.001	0.424	3.60	<0.001
NMS item 21 = 0	−2.23	1.04	0.149	−2.14	0.034
NMS item 5 = 0	−2.26	1.15	0.127	−1.97	0.051
BDI-II	0.15	0.08	0.109	1.83	0.070
NMS item 17 = 0	−2.22	1.28	0.097	−1.72	0.087
Education level (low)	−1.78	1.05	0.094	−1.70	0.091

* The independent variables were derived from the literature and included age, gender, education level (high/low), number of drugs per day, LEDD, disease duration, MDS-UPDRS III, H&Y, NMSQ, and BDI-II.

# The independent variables were NMSQ item 1 (hypersalivation), 5 (constipation), 13 (loss of interest), 15 (problems concentration), 17 (anxiety), and 21 (falls).

§ *The independent variables were NMSQ items 5 (constipation), 17 (anxiety), and 21 (falls) and LEDD, MDS-UPDRS III, BDI-II, age, education level (high/low), gender, and disease duration*.

Given the association between NMSQ and SAMS, we then aimed to determine which NMS (derived from NMSQ) were mainly associated with adherence. Analysis of NMSQ reported significant correlations between items 1 (hypersalivation; *r* = 0.18, *P* = 0.0035), 5 (constipation; *r* = 0.28, *P* = 0.001), 13 (loss of interest; *r* = 0.23, *P* = 0.007), 15 (problems in concentration; *r* = 0.20, *P* = 0.002), 17 (anxiety; *r* = 0.20, *P* = 0.0019), and 21 (falls; *r* = 0.21, *P* = 0.001) with SAMS. In the multiple stepwise regression analysis, we confirmed that items 5 (constipation), 17 (anxiety), and 21 (falls) predicted worse adherence to PD medication (corrected *R*^2^ = 0.14, *P* < 0.001, Table 2). These items also remained significant predictors after correction for LEDD, MDS-UPDRS III, BDI-II, age, education level, gender, and disease duration (corrected *R*^2^ = 0.23, *P* < 0.001, Table 2).

## Discussion

This study aimed to replicate the association between nonadherence and NMS in PD, which was recently demonstrated by Straka et al. (18). We discuss some methodological aspects first when comparing our results with that of Straka et al. In terms of age, gender, education level, MDS-UPDRS III, disease duration, and H&Y stage, our cohort was comparable to that of Straka et al., but our patients had a lower LEDD. We used different questionnaires. Using 18 or 8 items, both SAMS and MMAS-8 address self-reported nonadherent behavior. Categorization into adherent and nonadherent differs depending on the used cutoff value. Straka et al. used an MMAS-8 cutoff of 0 for full adherence, 1 and 2 points for moderate adherence, and ≥3 points for nonadherence. In their study, 33.9% reported a high level of adherence, 29.8% a medium level of adherence, and 36.3% a low level of adherence. In contrast, in our study, 10.9% were fully adherent, 73% moderately nonadherent, and 16.1% showed clinically significant nonadherence. The obtained prevalence differs because we used a different approach by using a study- and sample-specific cutoff for nonadherence. In line with many studies, we assumed that nonadherence becomes clinically significant when <80% of prescribed medication is taken (14, 25). Both SAMS and MMAS-8 cover forgetfulness and intentional nonadherence by modification of prescribed medication. Moreover, SAMS covers missing knowledge about prescribed medication as a crucial aspect for nonadherence (6, 26–29). Depression is an important risk factor for nonadherence (3, 4, 6, 10, 11, 15, 30); hence, this cofactor was assessed in our study with BDI-II and in the study by Straka et al. with GDS. Becks depression inventory II and GDS are both valid screening tools and have adequate accuracy to detect depression in PD (31). For detecting NMS burden, we used NMSQ, and Straka et al. used NMSS. Nonmotor symptoms questionnaire assesses for the presence of 30 common NMS using yes or no questions. In contrast, NMSS also detects the severity and frequency of NMS (32, 33). Moreover, both scores do not track the same NMS.

In line with earlier studies in PD, our multivariable analyses confirmed that depression, poor motor function (MDS-UPDRS III), lower education level, and higher LEDD are associated with nonadherence to medication (3, 4, 17). With reference to the study by Straka et al., we confirmed that the overall burden of NMS (NMSQ total score) is associated with nonadherence. However, we believe one has to be cautious when interpreting these results for the following reasons. In Straka's study, depression (assessed with GDS) was not a significant predictor of MMAS-8 when NMSS was entered into the model. This is surprising for two reasons. Although NMSS (as well as NMSQ) asks for depressive symptoms, one would expect that GDS questionnaire is more accurate in measuring depression. Therefore, we would assume that GDS should be associated with nonadherence just as BDI-II was associated with nonadherence in our study. This is because depression is a relevant predictor of nonadherence in many studies across different diseases and in PD (3, 4, 6, 10, 11, 15, 30). Our study, in principle, underlines that NMS burden is a predictor of adherence. This was also the case in our study after correction for other cofactors known to influence adherence, i.e., depression, motor impairment, and education level. However, we have to admit that after correction, the association between adherence and NMSQ was weak.

In addition, we and Straka et al. observed some correlations with distinct NMS items. Straka et al. found that excessive daytime sleepiness, anhedonia, and forgetfulness predict worse adherence. However, in our study, constipation, anxiety, and falls predicted worse adherence after correction for depression, education, and markers of disease severity. One can speculate about the reasons for these associations. While association to anxiety (related to depression or fear of drug side effects) and falls (related to later PD stages) seems somehow reasonable (34, 35), the association between constipation and adherence is difficult to explain. However, given the exploratory approach of both studies, one cannot rule out false positive associations between nonadherence and these specific NMS.

The study is not free of limitations. Patients with PD dementia were excluded. Therefore, the results cannot be generalized to all patients with PD. We also focused on self-reported and personal-related factors of nonadherence and therefore used a questionnaire to quantify adherence. For a holistic understanding of the association between NMS and adherence, it would be useful to incorporate additional methods to assess adherence (e.g., electronic pill monitoring). It is well-known that self-reports overestimate adherence compared with electronic monitoring (36). Thus, the reported prevalence of nonadherence has to be taken with caution.

## Conclusion

Our study, in principle, confirms the association between NMS burden and nonadherence in PD. However, we believe that the relevance of NMSQ for adherence is lower in contrast to other factors. More studies are necessary to explore the impact of distinct NMS on self-reported adherence.

## Data Availability Statement

The raw data supporting the conclusions of this article will be made available by the authors, without undue reservation upon reasonable request to the corresponding author.

## Ethics Statement

The studies involving human participants were reviewed and approved by Local Ethics Committee of the Jena University Hospital. The patients/participants provided their written informed consent to participate in this study.

## Author Contributions

SM and TP: contributed conception and design of the study and performed the statistical analysis. SM: organized the database and wrote the first draft of the manuscript. HZ: wrote sections of the manuscript. TP and OW: manuscript review and critique. All authors contributed to manuscript revision, read and approved the submitted version.

## Conflict of Interest

The authors declare that the research was conducted in the absence of any commercial or financial relationships that could be construed as a potential conflict of interest.

## Abbreviations

BDI: Becks depression inventory
H&Y: Hoehn and Yahr
LEDD: Levodopa equivalent daily doses
MDS-UPDRS: Movement Disorder Society-sponsored revision of the unified Parkinson's disease rating scale
NMSQ: Nonmotor symptoms questionnaire
PD: Parkinson's disease
SAMS: Stendal adherence to medication score.
